# Validation of the Five-Factor Self-Concept Questionnaire AF5 in Brazil: Testing Factor Structure and Measurement Invariance Across Language (Brazilian and Spanish), Gender, and Age

**DOI:** 10.3389/fpsyg.2018.02250

**Published:** 2018-11-20

**Authors:** Fernando Garcia, Isabel Martínez, Nekane Balluerka, Edie Cruise, Oscar F. Garcia, Emilia Serra

**Affiliations:** ^1^Department of Methodology of the Behavioral Sciences, University of Valencia, Valencia, Spain; ^2^Psychology Department, University of Castilla-La Mancha, Cuenca, Spain; ^3^Department of Social Psychology and Behavioral Sciences Methods, University of the Basque Country UPV/EHU, San Sebastian, Spain; ^4^Department of Economics and Social Work, University of Trier, Trier, Germany; ^5^Department of Developmental and Educational Psychology, University of Valencia, Valencia, Spain

**Keywords:** self-concept, multidimensional, adolescents, factorial invariance, multi-group analysis

## Abstract

Self-concept is widely conceptualized as multidimensional (Shavelson et al., 1976). The Five-Factor Self-Concept Questionnaire (AF5, García and Musitu, 2009) assesses five specific dimensions (i.e., academic, social, emotional, family, and physical). It is a psychometrically sound questionnaire, developed, and normed in Spain, which is widely used with Spanish-speaking samples. The validation of the AF5 in Brazil would expand its potential, and would facilitate cross-cultural research. To validate the Brazilian version of the AF5, the present study apply confirmatory factor analysis and multi-sample invariance analysis across sex (women vs. men), age (11–18 years old), and language (Brazilian [Portuguese] vs. Spanish). The sample consisted of 4,534 students (54.6%, women, 53.7%, Spanish) ranging in age from 11 to 18 years old (*M* = 14.61, *SD* = 2.09). The findings of the present study confirmed that the five-dimensional AF5 factorial structure provided the better fit to the data compared to alternative one-dimensional and orthogonal five-dimensional structures. The 30 items loaded appropriately on the five dimensions. Multi-group analysis for invariance between sex, age, and language groups showed equal loading in the five factors, equal covariation between the five dimensions, and equal error variances of items. Additionally, in order to obtain an external validity index, the five AF5 factors were related to both acceptance/involvement and strictness/imposition parenting dimensions. These results provide an adequate basis for meaningful comparative studies on a highly relevant construct, multidimensional self-concept, between male and female adolescents of different ages, and Brazilian (Portuguese) and Spanish-speaking samples. These results validate the instrument and confirm its suitability in cross-cultural research.

## Introduction

Self-concept is frequently defined as a person's self-perception formed through experiences with the environment (Kelley, 1973). This self-perception is influenced especially by evaluations of significant others, environmental reinforcements, and attributions for one's own behavior (see, Shavelson et al., 1976). Self-concept is generally considered both descriptive and evaluative (Shavelson et al., 1976; Marsh, 1993; Marsh and Craven, 2006; Swann et al., 2007; Marsh and O'Mara, 2008). While some models are based on the conceptualization of the self as a global evaluative component (self-esteem) (e.g., Rosenberg, 1979; Baumeister et al., 2003), the Shavelson et al. (1976) model integrates specific and global dimensions, so the global component integrates the specific components of self-concept (Marsh, 1993; Marsh et al., 2006). This multidimensional and hierarchical model proposed by Shavelson et al. (1976) has impacted self-concept research (Marsh and Hattie, 1996).

The AF5, Five-Factor Self-Concept Questionnaire (García and Musitu, 1999), based on the Shavelson et al. (1976) model, is one of the self-concept questionnaires most utilized in Spanish-speaking samples (e.g., Goñi-Grandmontagne et al., 2004; Bustos et al., 2015). The AF5 was developed, validated, and normed in Spain on a large sample of nearly 6,500 participants ranging in age from 10 to 62 years, providing national norms for sex and age. The questionnaire evaluates five self-concept dimensions (academic, social, emotional, family, and physical) that represent different qualities that are differentially related to distinct areas of human behavior (Shavelson et al., 1976; Marsh and O'Mara, 2008). The five dimensions examined in the AF5 questionnaire include, (i) academic or work self-concept, which refers to the perception the subjects have of the quality of their performance as a student or worker; (ii) social self-concept, which reflects the perceptions the subjects have of their performance in social relationships; (iii) emotional self-concept, which captures perception of the individual's own emotional state and responses to concrete situations; (iv) family self-concept, which reflects the subject's perception of their involvement, participation, and integration in the family setting, and; (v) physical self-concept, which consists in the person perception of their physical appearance and physical performance (García and Musitu, 1999; García et al., 2011).

Studies with the AF5 questionnaire reinforced a theoretical framework of self-concept based on the multidimensional perspective (Marsh and O'Mara, 2008). For example, although Baumeister et al. (2003) adopting a unidimensional perspective of the self-concept construct (p. 7), noted that “the modest correlations between self-esteem and school performance do not indicate that high self-esteem leads to good performance,” Fuentes et al. (2011a), found a correlation of .60 (*r*^2^ = 36%) between academic-AF5 self-concept and grade point average. In the same way, Gorostiaga et al. (2011) found that teenagers with high social-AF5 self-concept showed a higher level of emotional intelligence (specifically, a higher level of clarity of emotions and mood repair) than teenagers with low social-AF5 self-concept.

In general, the factor validity evidence of the AF5 is supported. Exploratory factor analyses were applied with Spanish (García and Musitu, 2009), Brazilian (Martínez et al., 2003), Mexican (Salum-Fares et al., 2011), and Italian samples (Marchetti, 1997). Confirmatory factor analyses reported validity evidence of the AF5 structure in samples from Spain (Tomás and Oliver, 2004; García et al., 2011; Murgui et al., 2012), United States (García et al., 2013), Peru (Bustos et al., 2015), Chile (García et al., 2011), Portugal (García et al., 2006), Basque Country (Elosua and Muñiz, 2010), and Catalonia (Cerrato et al., 2011). All these studies reported that all AF5 items loaded onto their corresponding theoretical subscales and that there were no complex items. The AF5 scale does not show presence of method effects due to negative wording items (Tomás and Oliver, 2004; García et al., 2011). The median of reliability estimates for the AF5 subscale scores in the literature ranged from 0.71 to 0.87, providing adequate evidence for the internal consistency of the subscales (Martínez et al., 2003; García and Gracia, 2009; Fuentes et al., 2011a,b; Table 1).

**Table 1:** Fifteen studies by country-, age-, and size-sample, and internal consistency (cronbach's reliability) in the five AF5 dimensions.

**Study**	**Country**	**Age**	***N***	**Academic**	**Social**	**Emotional**	**Family**	**Physical**
García and Musitu, 1999	Spain	10–62	6483	0.88	0.70	0.73	0.77	0.74
Martínez et al., 2003	Brazil	10–18	2142	0.82	0.53	0.69	0.71	0.73
Tomás and Oliver, 2004	Spain	10–60	5943	0.88	0.70	0.77	0.77	0.75
García et al., 2006	Portugal	18–62	1058	0.87	0.80	0.77	0.76	0.78
Musitu et al., 2007	Spain	12–17	1039	0.84	0.71	0.79	0.80	0.75
García and Gracia, 2009	Spain	12–17	1416	0.89	0.71	0.70	0.85	0.74
Fuentes et al., 2011a	Spain	12–17	1281	0.89	0.68	0.70	0.85	0.74
Fuentes et al., 2011b	Spain	12–17	632	0.91	0.82	0.74	0.88	0.80
García et al., 2013	US	14–18	624	0.86	0.74	0.78	0.87	0.73
Bustos et al., 2015	Peru	19–35	527	0.81	0.73	0.82	0.76	0.75
Garcia et al., 2018	Spain	12–75	1098	0.86	0.75	0.74	0.79	0.79
Riquelme et al., 2018	Spain	12–17	1445	—	—	0.71	0.85	0.76
Martínez et al., 2018	Spain (university videogamers)	20–29	490	0.77	0.77	0.81	—	0.78
Maiz and Balluerka, 2018	Spain (children)	8–11	464	0.82	0.56	0.72	0.65	0.69
	Spain (adolescents)	12–16	367	0.90	0.69	0.78	0.78	0.75
Martínez et al., 2019	Spain	12–17	1109	0.88	0.70	0.73	0.81	0.75
Median				0.87	0.71	0.74	0.79	0.75

Studies of the associations of the AF5 dimensions with related constructs showed theoretically interpretable relations. For example, recent studies on physical and exercise domains carried out with community samples of Spanish adolescents, evidenced that gender stereotypes, body image, and sport practice showed different relations with academic, physical, emotional and family self-concept (Mendo-Lázaro et al., 2017); moreover, physical activity during adolescence improves physical self-concept, integration into peer groups and academic results (Martínez and Hernández, 2017); sampling young adult Chilean judo-practitioners, it was revealed that motivational climate was related to physical self-concept and satisfaction with the task (Ortega et al., 2017). Clinical studies have shown, in a children and adolescents community sample, that food neophobia presented different associations with social, physical, and academic self-concept (Maiz and Balluerka, 2018); furthermore, an emotional intelligence program for women with breast cancer showed general increase on the five AF5 self-concept factor scores and a decrease in anxiety (Cejudo et al., 2017). Studies on adolescent problems with community samples showed that low emotional, family, and physical self-concept are associated with initiation into substance use during early adolescence (Riquelme et al., 2018); that more vulnerable adolescent victims of gender-based violence have the lowest emotional and physical self-concept (Abilleira and Rodicio-García, 2017); and that lastly, in analyzing school violence, adolescents with high levels of participation in the community obtained high scores on academic and social self-concept and on satisfaction with life, and low scores on loneliness (Crespo-Ramos et al., 2017). From the positive psychology perspective, in a community sample of adolescents, it was revealed that the most contributing factor to increase the subjective well-being is family self-concept (González-Carrasco et al., 2017). Finally, parenting studies analyzing the influence of parental practices on self-concept in Spain (Fuentes et al., 2015b; Riquelme et al., 2018), other European (Calafat et al., 2014) and Latin-American countries (Peru, Bustos et al., 2015; Carranza and Bermúdez-Jaimes, 2017; Brazil, Martínez et al., 2007; Martínez and García, 2008), and also in the United States (García et al., 2013), have shown that parenting characterized by the use of acceptance and involvement practices is associated with higher levels of self-concept, in several dimensions, than parenting characterized by the use of practices of strictness and imposition (Fuentes et al., 2011a,b; Martínez-González et al., 2016; Martínez et al., 2017, 2019).

Additionally, the AF5 scale has served as criteria to validate self-concept (Garaigordobil and Aliri, 2011; Goñi et al., 2011; Vera and Nieto, 2018) and self-esteem instruments (Martín-Albo et al., 2007a). It has also been utilized as criteria to validate scales of related measures, such as parental socialization (Martínez et al., 2017), effective personality (Pellerano et al., 2006), social skills (Miranda-Zapata et al., 2014), sport motivation (Martin-Albo et al., 2007b), academic motivation (Nuñez et al., 2010), and peer mentoring (Alonso et al., 2010).

The AF5 also has been applied in the Portuguese language. For example, it has been used to analyze the impact of intervention programs for adolescent students in Portugal, (Coelho et al., 2014, 2016, 2017), students disabilities (Valenzuela-Zambrano et al., 2016), and optimal parenting style (Rodrigues et al., 2013). In Brazil, the AF5 scale has been applied mainly in parenting research, studying optimal parenting styles (Martínez et al., 2003, 2007; Martínez and García, 2008). However, there is a main gap in literature since the AF5 still has not been validated in Brazilian (Portuguese) language. There is only a validation study in Portugal sampling adults (García et al., 2006).

The aim of the present study is to test the factor structure of the AF5 across sex, adolescent age, and Brazilian and Spanish languages. For the validation process, we followed a sequential main two-step method. First, we examined the fit of the correlated five-factor model of the AF5 structure (García and Musitu, 2009) compared to one-dimensional and five-dimensional orthogonal competitive models. Next, we tested the factorial invariance of the AF5 factor structure for language samples (Brazilian [Portuguese] vs. Spanish), sex (men vs. women), and adolescent age (11–12, 13–14, 15–16, and 17–18 years old). Following the theoretical structure (Shavelson et al., 1976; García and Musitu, 2009) and previous studies (Tomás and Oliver, 2004; García et al., 2011, 2013; Murgui et al., 2012; Bustos et al., 2015), we hypothesize that: (1) the five-factor AF5 correlated model would fit the data better than competitive models; and (2) language groups, gender, and adolescent age would be invariant with respect to the hypothesized AF5 correlated structure.

Additionally, to obtain an external validity index, the five AF5 self-concept dimensions will be related with parental socialization practices, a variable classically related with self-concept (Felson and Zielinsky, 1989; Barber, 1990; Musitu and García, 2001, 2004). Parenting dimensions of acceptance/involvement and strictness/imposition are considered. According to previous research (Musitu and García, 2001; Fuentes et al., 2011a,b; Martínez et al., 2017) it is expected that self-concept dimensions will be related positively with parental practices of acceptance/involvement and negatively with parental practices of strictness/imposition.

## Methods

### Participants

The sample was composed of 4,534 students (54.6% being women, 53.7% being Spanish) covering the adolescent age range (age range = 11–18 years old; *M* = 14.61, *SD* = 2.09) (see Table 2).

**Table 2:** Sample distribution by language, sex, and age.

		**Academic**			**Social**			**Emotional**			**Family**			**Physical**		
	***N***	***M (SD)***	***Skew***	**α**	***M (SD)***	***Skew***	**α**	***M (SD)***	***Skew***	**α**	***M (SD)***	***Skew***	**α**	***M (SD)***	***Skew***	**α**
Spanish	2437	368.8(114.9)	−0.43	0.90	460.7(088.1)	−1.36	0.85	316.6(114.1)	−0.05	0.76	496.3(096.3)	−1.91	0.85	348.4(128.7)	−0.40	0.85
Brazilian	2097	425.9(108.6)	−0.79	0.84	472.5(096.3)	−1.73	0.85	297.1(114.1)	−0.12	0.73	494.1(098.5)	−1.86	0.83	394.7(114.6)	−0.62	0.73
Men	2058	383.2(113.2)	−0.48	0.86	462.7(089.6)	−1.52	0.83	332.5(108.4)	−0.15	0.72	499.4(088.0)	−1.97	0.81	395.9(116.0)	−0.75	0.80
Women	2476	405.2(116.6)	−0.65	0.89	469.1(094.2)	−1.55	0.86	286.9(115.3)	0.02	0.75	491.8(104.4)	−1.79	0.86	348.2(127.2)	−0.34	0.80
11–12	866	425.0(116.1)	−0.77	0.86	477.5(100.8)	−1.87	0.86	315.7(125.2)	−0.15	0.75	517.7(092.5)	−2.53	0.84	395.9(129.5)	−0.71	0.80
13–14	1281	384.7(126.3)	−0.59	0.89	463.1(093.5)	−1.47	0.84	306.2(113.2)	−0.10	0.74	495.4(096.3)	−1.96	0.83	367.6(125.4)	−0.55	0.81
15–16	1403	388.0(107.6)	−0.42	0.87	464.0(086.2)	−1.44	0.84	308.5(112.3)	−0.12	0.76	489.7(098.6)	−1.77	0.85	366.3(120.4)	−0.55	0.81
17–18	984	393.0(107.1)	−0.52	0.87	463.4(090.1)	−1.40	0.85	300.9(109.2)	0.04	0.74	483.2(097.9)	−1.60	0.84	354.8(121.5)	−0.36	0.80

### Procedure

The sample frame of the present study was adolescents from secondary schools from large metropolitan areas (with over one million inhabitants in each area) on the East Coast of Spain and in Northeast Brazil. The data was collected from 26 educational centers (14, Spanish and 12, Brazilian) chosen through the simple random sampling method from a complete list of centers. An a priori analysis was computed to calculate the minimum sample size that was required in order to recover the population factor structure (Guadagnoli and Velicer, 1988; Gracia et al., 1995; Pérez et al., 1999; García et al., 2008). We fixed the average discrepancy at least as small as .05 between the population parameter and the estimated sample values of factor loadings with an average target loading of 0.5 on a factor (García and Musitu, 2009), obtaining a minimum sample size of 625 subjects (Formula 3; Guadagnoli and Velicer, 1988). The minor sample size of 866 (11–12 years old, Table 2) showed an average discrepancy of 0.04 (Guadagnoli and Velicer, 1988).

The research protocol was approved by the Research Ethics Committee of the Program for the Promotion of Scientific Research, Technological Development and Innovation of the Spanish Valencian Region, which supported this research. First, we obtained permission to conduct this study from the Research and Evaluation Board of the Public-School Board in secondary schools in the cities where the data collection took place. Second, we were required to obtain permission from the individual heads of each center. After each head of center granted us permission, the individual teachers allowed for the administration of the questionnaires during their class time. Finally, we provided a detailed description of our study to all parents and guardians of the students who were to potentially participate in our research in order to fully inform them of the questionnaires their child would be asked to complete. A parent or guardian for all minor participants then gave us express written consent for their child to participate in our study. Additionally, each student also signed an assent form stating that their participation in our study was completely voluntary. The researchers only administered the questionnaires to the students who had agreed to voluntarily participate as well as had written parental consent on file with our research team to do so. All the questionnaires were completed anonymously. The questionnaires were examined for questionable response patterns, such as reporting implausible inconsistencies between negatively and positively worded responses or “maximum-scale” behavior on responses (Tomás and Oliver, 2004; García et al., 2011). About 2% (*n* = 90) of the cases were identified as questionable and removed from the sample.

### Instruments

The AF5 (García and Musitu, 2009) questionnaire was designed to measure five self-concept dimensions: academic (e.g., “I do my homework well”), social (e.g., “I am a friendly person”), emotional (e.g., reversed item, “Many things make me nervous”), family (e.g., “I feel that my parents love me”), and physical (e.g., “I like the way I look”). The scale consists of 30 items, six for each dimension. The items are statements that the participant must rate using a continuous response on a 99-point scale (visualized as a thermometer), ranging from 1: complete disagreement, to 99: complete agreement. Table 2 shows the descriptive statistics for each subscale and each group.

To translate the AF5 from the original version (Spanish) into Brazilian (Portuguese), we used the back-translation method (Brislin, 1970) to achieve concept equivalence (i.e., the items were comparable to other language versions of the scale). After obtaining authorization from the scale's authors, the original instrument was translated into Brazilian (Portuguese) from Spanish by three bilingual colleagues, selected for their proficiency in Spanish and the Portuguese language. They carried out a cross-check on item grammar, clarity, and content equivalence. Then, an independent, bilingual researcher back-translated the Portuguese items into Spanish, which were then submitted for a final examination by the authors (García et al., 2006; Martínez et al., 2011; Magallares and Talo, 2016).

The Parental Socialization Scale ESPA29 (Musitu and García, 2001) measures different socialization practices in response to 29 situations representative of everyday family life. The respondents rate their father's and mother's practices separately using a 4-point scale, 1 “never,” 2 “sometimes,” 3 “most times,” and 4 “always.” The 29 scenarios are divided into 13 that refer to situations of obedience in which the child acts in accordance with the family norms (e.g., “If the school reports that I am well-behaved”) and 16 refer to situations of disobedience in which the child does not conform to family norms (e.g., “If I leave home to go somewhere without asking anyone for permission”). In the 13 situations of obedience the practices of warmth (“He/she shows affection”) and indifference (“He/she seems indifferent) are evaluated. In the 16 situations of disobedience the practices of reasoning (“He/she talks to me”), detachment (It's the same to him/her”), verbal scolding (“He/she scolds me”), physical punishment (“He/she hits me”), and revoking privileges (“He/she takes something away from me”) are rated. The acceptance/involvement dimension score is calculated through the average of scores for the warmth, reasoning, indifference, and detachment subscales (the indifference and detachment subscales are inverted since they are inversely related to the dimension). The score for the strictness/imposition dimension is obtained through the average of the scores for the revoking privileges, verbal scolding, and physical punishment subscales. The ESPA29 theoretical structure was confirmed in studies conducted in Spain (Musitu and García, 2001), Brazil (Martínez et al., 2011, 2012) and the United States (Martínez et al., 2017) showing an invariant pattern for adolescent males and females. This scale has been utilized in a great many studies to consistently relate parenting with other variables (e.g., Martínez and García, 2007; Gracia et al., 2012; Martínez et al., 2013; Fuentes et al., 2015a,b). It is remarkable that the ESPA29 parenting acceptance/involvement dimension has been related to high adolescents' self-concept, and the strictness/imposition dimension has been related to low adolescent self-concept (e.g., Fuentes et al., 2011a,b; García and Gracia, 2014).

### Data analysis

**Figure 1:**
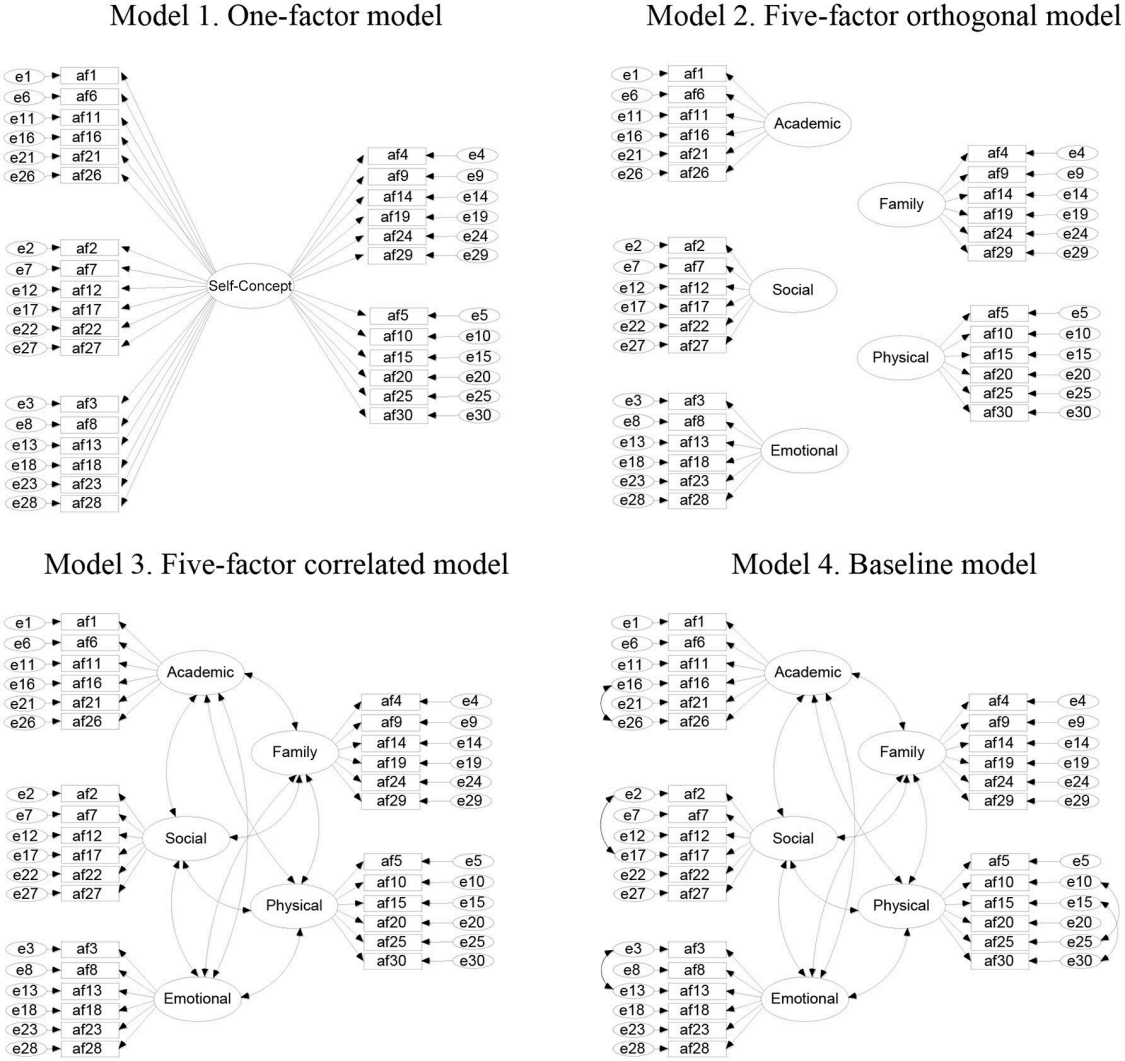
The four competitive models. Spanish sample freed error covariances of Model 4: 16–26, 2–17, 3–13, 10–25, and 15–30; Brazilian, sex and adolescent age samples: 10–25, and 15–30.

In order to test the first hypothesis, we compared the fit of the hypothesized five-factor correlated model with the fit of other competitive models separately for each group by language, sex, and age (see Figure 1). First, a one-factor model was tested. This model portrays self-concept as a one-dimensional construct (e.g., Rosenberg, 1965; Baumeister et al., 2003). Next, we tested an orthogonal five-factor model. This model looks at self-concept as a multidimensional construct considering the five AF5 dimensions as orthogonal (non-related) dimensions underlying self-concept (Burbach and Bridgemen, 1976; Shavelson et al., 1976; García et al., 2011, 2013). Lastly, the correlated five-factor model based on the AF5 was tested (Shavelson et al., 1976; Byrne and Shavelson, 1996; García and Musitu, 2009). In the fourth and final model, we freed error covariances for the strongly correlated pairs of items within each factor of the third model (Byrne and Shavelson, 1996; Tomás and Oliver, 2004; García et al., 2006).

In line with preliminary studies (Tomás and Oliver, 2004; García et al., 2006), we used maximum likelihood (ML) as the method of estimation in the confirmatory factor analyses (West et al., 1995). This method assumes that variables have a multivariate normal distribution. However, non-normality seems to have little impact on model parameters which were estimated using ML (i.e., parameters remain relatively unbiased); either way, it always reduces the confirmatory fit index measures (West et al., 1995; Tomás and Oliver, 2004; Tomas et al., 2010). In this study, a large sample was used in order to adequately control the sample error size (with a small sample size, robust statistics may be more appropriate). We applied structural equation models (SEMs) to examine adjustment of the models to the data. SEMs were calculated with EQS 6.1 (Bentler, 1995) using the maximum likelihood robust estimation method, due to the deviation of the multinormal data (all Mardia's normalized coefficient > 50, *p* < 0.01). Overall, chi-square tests of goodness-of-fit models are likely to be significant given that the chi-square statistic is overly sensitive to sample size (e.g., Bentler and Bonett, 1980; Cheung and Rensvold, 2002; García et al., 2006). Therefore, other fit indexes were calculated: χ2/gl, a score of 2.00–3.00 or lower indicates a good fit; root mean squared error of approximation (RMSEA), values lower than 0.08 are considered acceptable; normed fit index and comparative fit index, NFI and CFI, whose value must exceed 0.90; and the information criterion of Akaike, AIC (Akaike information criterion), where the lowest value indicates the highest parsimony (Akaike, 1987) (see Table 3 above). The estimation method was maximum likelihood (ML), which, although assuming multivariate normality, is reasonably robust to its non-compliance (Curran et al., 1996). The criteria used are in line with those proposed by Hu and Bentler (1999) and Kline (1998), and are what is typically utilized in this type of analysis (West et al., 1995; Tomás and Oliver, 2004; García et al., 2006, 2011; Garcia et al., 2018; Tomas et al., 2010; Mayordomo-Rodríguez et al., 2015).

**Table 3:** Goodness-of-fit indicator models by language, sex, and age.

**Model**	**S-B χ^2^**	***df***	**RMSEA [90% CI]**	**CFI**	**ΔCFI**	**AIC**
**SPANISH**						
Model 4^$^	1526.58	390	0.035[0.033–0.036]	0.951	0.032	746.58
Model 3	2270.81	395	0.044[0.042–0.046]	0.919	0.043	1480.81
Model 2	3262.82	405	0.054[0.052–0.056]	0.876	0.502	2452.82
Model 1	14859.28	405	0.121[0.119–0.123]	0.374		14049.28
**BRAZILIAN**						
Model 4^#^	1051.52	393	0.028[0.026–0.030]	0.951	0.018	265.52
Model 3	1314.38	395	0.033[0.031–0.035]	0.932	0.097	524.38
Model 2	2627.78	405	0.051[0.049–0.053]	0.835	0.314	1817.78
Model 1	6857.50	405	0.087[0.085–0.089]	0.521		6047.50
**MEN**						
Model 4^#^	1156.06	393	0.031[0.029–0.033]	0.947	0.016	370.06
Model 3	1393.18	395	0.035[0.033–0.037]	0.931	0.073	603.18
Model 2	2474.52	405	0.050[0.048–0.051]	0.858	0.396	1664.52
Model 1	8152.67	405	0.096[0.095–0.098]	0.462		7342.67
**WOMEN**						
Model 4^#^	1402.06	393	0.032[0.030–0.034]	0.952	0.017	616.06
Model 3	1767.77	395	0.037[0.036–0.039]	0.935	0.063	977.77
Model 2	3101.20	405	0.052[0.050–0.054]	0.872	0.408	2291.2
Model 1	11692.51	405	0.106[0.104–0.108]	0.464		10882.51
**11–12 YEARS OLD**						
Model 4^#^	672.21	393	0.029[0.025–0.032]	0.947	0.003	−113.79
Model 3	720.11	395	0.031[0.027–0.034]	0.944	0.105	−69.89
Model 2	1341.68	405	0.052[0.049–0.055]	0.839	0.326	531.68
Model 1	3237.12	405	0.090[0.087–0.093]	0.513		2427.12
**13-14 YEARS OLD**						
Model 4^#^	845.88	393	0.030[0.027–0.033]	0.955	0.017	59.88
Model 3	1016.06	395	0.035[0.032–0.038]	0.938	0.060	226.06
Model 2	1640.83	405	0.049[0.046–0.051]	0.878	0.428	830.83
Model 1	5953.86	405	0.103[0.101–0.106]	0.450		5143.86
**15–16 YEARS OLD**						
Model 4^#^	1087.01	393	0.035 [0.033–0.038]	0.941	0.026	301.01
Model 3	1393.40	395	0.042 [0.040–0.045]	0.915	0.043	603.40
Model 2	1916.86	405	0.052 [0.049–0.054]	0.872	0.486	1106.86
Model 1	7627.00	405	0.113 [0.111–0.115]	0.386		6817.00
**17–18 YEARS OLD**						
Model 4^#^	933.90	393	0.037 [0.034–0.040]	0.928	0.018	147.90
Model 3	1145.28	395	0.044 [0.041–0.047]	0.910	0.058	355.28
Model 2	1638.17	405	0.056 [0.053–0.058]	0.852	0.393	828.17
Model 1	4908.79	405	0.106 [0.104–0.109]	0.459		4098.79

*Satorra-Bentler Chi-square tests statistically significant (p < 0.01). df, degrees of freedom; RMSEA, Root Mean Squared Error of Approximation; CFI, Comparative Fit Index; AIC, Akaike Information Criterion (computed as χ^2^ – 2df)*.

$ *Freed error covariances: 16–26, 2–17, 3-13, 10–25, and 15–30*.

# *Freed error covariances: 10–25, and 15–30*.

In order to test the second hypothesis, we compared four nested models across samples of language, sex, and adolescent age. All the previous analyses were conducted for each sample separately. However, once the baseline model was established with each sample, we tested if the CFA model fit each language, sex, and adolescent age samples well. We conducted the following sequence of increasingly more restrictive tests of invariance across each related sample: (a) unconstrained, without any restrictions across parameters, (b) factor pattern coefficients, (c) factor variances and covariances, and, (d) equality of the error variances.

To determine whether constraining parameters are invariant across groups yielding a meaningful decrease in fit, the Δχ^2^ has traditionally been used as the index of difference in fit (e.g., Spencer et al., 2005). However, due to its sensitivity to sample size, the use of Δχ^2^ has been criticized (Kelloway, 1995; Cheung and Rensvold, 2002). Cheung and Rensvold (2002) provided evidence that the ΔCFI was robust for testing the multi-group invariance. On the basis of extensive simulations, they also determined that an absolute ΔCFI value higher than 0.01 was indicative of a meaningful fall in fit. If the ΔCFI indicated that the constrained model did not lead to a meaningful decrease in fit as compared to the unconstrained model, the constrained parameters were considered to be invariant across groups.

Furthermore, the AF5 scale's dimensions were related to main parenting socialization practices of acceptance/involvement and strictness/imposition, which was measured through the ESPA29 instrument (García and Musitu, 1999), using confidence intervals around Pearson *r*'s (Balluerka et al., 2009; Gorostiaga et al., 2011; Cava et al., 2013; Martínez et al., 2017).

## Results

### Confirmatory factor analysis in each sample

Fit indexes for the four competitive models in each sample are reported in Table 3. As expected, when Model 4 was applied to each sample, all indexes achieved better fit, and when Model 1 was applied, all indexes achieved poorer fit. For example, in the analysis of the Spanish language sample (Table 3), in the first step (Model 1) we constrained the data to be consistent with the single one-factor model. With this model, statistics generally failed to meet conventional standards (RMSEA, 0.12, CFI, 0.37, and, AIC, 14049), indicating a very poor fit. In the second step (Model 2), we constrained data to the five-factor model proposed by the AF5 structure, but regarding dimensions as orthogonal. This model provided considerable increase of fit with respect to the previous one-factor model (RMSEA, 0.05 [no overlapping 90% upper-CI of first model: 0.12], CFI, 0.88, and, AIC, 2453). In the third step (Model 3), we examined the same five-factor model but with five correlated dimensions, which resulted in improved fit (RMSEA, 0.04 [no overlapping 90% upper-CI of second model: 0.06], CFI, 0.92, and, AIC, 1481) as compared to the orthogonal model. Finally, in the last step (Model 4), we freed error covariances for the strongly correlated item pairs in each factor of the third model. This model provided another increase of fit (RMSEA, 0.04 [no overlapping 90% upper-CI of third model: 0.05], CFI, 0.95, and, AIC, 747) compared to Model 3. Overall, the results obtained through separately conducted analyses for the language, sex, and age samples, indicated support for the AF5 correlated model and produced a better fit than all competitive models.

### Multi-sample confirmatory factor analysis of invariance across related samples

Fit indices of the four increasingly restricted nested models of invariance across related samples (language, sex, and age) are reported in Table 4. As expected, the unconstrained model A (consisting of the baseline Model 4 for each of the two language samples, each sex sample, and each of the four adolescent age samples) suggested a common factor structure across all related analyzed samples. According to expectations, the constrained model B (constraining the pattern coefficients across the related samples) resulted in continued good fit, suggesting that factor loadings were invariant across all the related analyzed samples. As was expected, the constrained model C (constraining the pattern structural variances and covariances across the related samples) resulted in continued good fit, suggesting no differences in structural variances and covariances across all related analyzed samples. Finally, the constrained model D (constraining the error variances across the related samples) resulted in no changes in goodness-of-fit across sex samples (men vs. women). Regarding language and adolescent age samples, only partial differences were found in error variances. For example, in the analysis of the Spanish vs. Brazilian (Portuguese) language samples (Table 4), in the first step, the unconstrained model (consisting of the baseline Model 4 of both language samples) showed a good fit (RMSEA, 0.02, CFI, 0.95, and, AIC, 1006), suggesting a common factor structure across the two language samples. In the second step, constraining the pattern of factor coefficients across both language samples resulted in continued good fit, |ΔCFI| < 0.01 and RMSEA, 0.02 overlapping with 90% lower-CI of model A: 0.02. In the third step, constraining the pattern structural variances and covariances of both samples resulted in continued good fit, |ΔCFI| < 0.01 and RMSEA, 0.03 overlapping with 90% lower-CI of model B: 0.03. In the fourth step, only partially constraining the error variances (see note at the end of Table 4) resulted in no changes in goodness-of-fit, |ΔCFI| < 0.01 and RMSEA, 0.03 overlapping with 90% lower-CI of model C: 0.03.

**Table 4:** Goodness-of-fit indicator models of multi-sample analysis for the invariance across language, sex, and age.

**Model**	**S-B χ^2^**	***df***	**RMSEA [90% CI]**	**CFI**	**ΔCFI**	**AIC**
**LANGUAGE**						
Model A	2571.55	783	0.022[0.021–0.023]	0.951		1005.55
Model B	2746.37	808	0.023[0.022–0.024]	0.947	−0.004	1130.37
Model C	2974.47	823	0.024[0.023–0.025]	0.941	−0.006	1328.47
Model D^$^	3288.09	846	0.025[0.024–0.026]	0.932	−0.009	1596.09
**SEX**						
Model A	2558.59	786	0.022[0.021–0.023]	0.950		986.59
Model B	2617.99	811	0.022[0.021–0.023]	0.949	−0.001	995.99
Model C	2746.27	826	0.023[0.022–0.024]	0.946	−0.003	1094.27
Model D	2898.80	856	0.023[0.022–0.024]	0.943	−0.003	1186.80
**AGE**						
Model A	3522.06	1572	0.017[0.016–0.017]	0.945		378.06
Model B	3639.27	1647	0.016[0.016–0.017]	0.941	−0.004	345.27
Model C	4771.71	1692	0.017[0.016–0.017]	0.941	0.000	1387.71
Model D^#^	4109.28	1776	0.017[0.016–0.018]	0.932	−0.009	557.28

*Satorra-Bentler Chi-square tests statistically significant (p < 0.01). df, degrees of freedom; RMSEA, Root Mean Squared Error of Approximation; CFI, Comparative Fit Index; AIC, Akaike Information Criterion (computed as χ^2^ – 2df)*.

$ *Freed restriction of same multi-sample error covariance: 1, 2, 6, 11, 21, 25, and 30*.

# *Freed restriction of same multi-sample error covariance: 7, and 16*.

Tables 5, 6 give an overview of the parameters of the most constrained model. Invariance testing across language, sex, and adolescent age showed that the correlated five-factor model operates in a similar way for all the analyzed samples.

**Table 5:** Parameter estimates (and standard errors) of load and errors for three multi-sample confirmatory factor analysis model.

		**Factor loadings**			**Errors**		
**Factor**	**Item**	**Language**	**Sex**	**Age**	**Language**	**Sex**	**Age**
AC	1	0.65(0.00)	0.70(0.00)	0.70(0.00)	^1^	241.6(05.8)	242,1(05,8)
	6	0.78(0.03)	0.82(0.03)	0.82(0.03)	^2^	215.2(06.1)	213,6(06,1)
	11	0.66(0.03)	0.67(0.03)	0.67(0.03)	^3^	326.6(07.6)	321,7(07,5)
	16	0.62(0.03)	0.67(0.03)	0.72(0.03)	352.1(08.1)	336.8(07.9)	^8^
	21	0.74(0.03)	0.78(0.03)	0.78(0.03)	^4^	242.8(06.3)	243,6(06,3)
	26	0.76(0.03)	0.78(0.03)	0.78(0.03)	249.4(06.4)	237.2(06.2)	240,2(06,2)
SO	2	0.74(0.00)	0.76(0.00)	0.77(0.00)	^5^	158.9(04.3)	157,6(04,3)
	7	0.70(0.02)	0.71(0.02)	0.76(0.02)	182.5(04.5)	180.7(04.5)	^9^
	12	0.73(0.02)	0.72(0.02)	0.72(0.02)	217.7(05.5)	221.8(05.7)	222,1(05,7)
	17	0.72(0.02)	0.71(0.02)	0.70(0.02)	176.0(04.6)	184.2(04.6)	185,8(04,6)
	22	0.59(0.02)	0.59(0.02)	0.58(0.02)	324.2(07.4)	325.0(07.4)	325,2(07,4)
	27	0.69(0.02)	0.70(0.02)	0.69(0.02)	220.1(05.3)	217.5(05.4)	216,8(05,4)
EM	3	0.45(0.00)	0.47(0.00)	0.49(0.00)	546.1(12.6)	527.8(12.3)	525,5(12,3)
	8	0.62(0.06)	0.61(0.06)	0.62(0.05)	492.5(13.2)	493.4(13.1)	493,6(13,1)
	13	0.55(0.05)	0.56(0.06)	0.58(0.05)	585.8(14.5)	554.4(13.9)	556,6(14,1)
	18	0.56(0.06)	0.56(0.06)	0.56(0.05)	574.8(14.4)	575.6(14.4)	578,4(14,3)
	23	0.55(0.06)	0.52(0.05)	0.53(0.05)	583.0(14.4)	593.5(14.3)	594,3(14,4)
	28	0.67(0.07)	0.65(0.06)	0.66(0.06)	510.6(15.1)	528.7(15.0)	530,2(14,9)
FA	4	0.60(0.00)	0.60(0.00)	0.59(0.00)	399.0(09.2)	398.4(09.2)	396,7(09,2)
	9	0.71(0.03)	0.71(0.03)	0.70(0.03)	221.1(05.6)	220.5(05.6)	220,6(05,6)
	14	0.69(0.03)	0.69(0.03)	.069(0.03)	261.1(06.5)	260.9(06.5)	261,6(06,5)
	19	0.70(0.03)	0.70(0.03)	0.70(0.03)	207.8(05.2)	209.6(05.2)	208,4(05,2)
	24	0.73(0.03)	0.73(0.03)	0.73(0.03)	244.6(06.3)	245.8(06.4)	245,8(06,4)
	29	0.74(0.02)	0.74(0.02)	0.74(0.02)	143.3(03.8)	144.0(03.8)	143,8(03,8)
PH	5	0.69(0.00)	0.70(0.00)	0.69(0.00)	353.8(09.8)	350.7(09.9)	353,6(09,9)
	10	0.53(0.03)	0.54(0.03)	0.54(0.03)	763.2(18.1)	721.1(17.2)	750,3(17,9)
	15	0.57(0.03)	0.59(0.03)	0.59(0.03)	510.6(12.5)	516.6(12.9)	513,0(12,9)
	20	0.64(0.03)	0.63(0.03)	0.64(0.03)	512.9(13.2)	528.3(13.4)	524,0(13,5)
	25	0.61(0.03)	0.62(0.03)	0.62(0.03)	^6^	503.5(12.7)	515,6(13,0)
	30	0.59(0.03)	0.63(0.03)	0.64(0.03)	^7^	495.0(12.8)	485,7(12,8)

*AC, Academic; SO, Social; EM, Emotional; FA, Family; PH, Physical. All estimated parameters were statistically significant for α = 0.05. Negatively worded items (3, 4, 8, 12, 13, 14, 18, 22, 23, and 28) were inverted*.

*^1-7^Spanish/Brazilian: ^1^172.2 (5.7)/ 313.1 (10.8), ^2^157.3 (6.3)/ 276.0 (10.9), ^3^227.7 (7.6)/ 390.9 (13.6), ^4^178.5 (6.5)/ 304.0 (11.3), ^5^112.6 (4.8)/ 191.5 (7.2), ^6^397.5 (14.2)/ 640.1 (23.0), ^7^365.0 (13.4)/ 604.8 (21.7)*.

*^8-9^11–12/ 13–14/ 15–16/ 17–18 years old: ^1^448.5 (23.2)/ 363.4 (15.7) / 294.3 (12.4) / 251.9 (12.9), ^2^229.8 (12.4)/ 221.3 (9.9) / 141.6 (6.5) / 131.290 (7.3)*.

**Table 6:** Parameter estimates (and standard errors) of factor variances, covariances, and [correlations] for three multi-sample confirmatory factor analysis model.

	**AC**	**SO**	**EM**	**FA**	**PH**
**LANGUAGE**					
AC	226.9 (08.6)	81.2 (04.3)	−14.7 (03.3)	95.8 (04.8)	116.4 (05.8)
SO	[0.36]	228.2 (07.7)	−2.6 (03.3)	86.1 (04.6)	133.8 (05.9)
EM	[−0.08]	[−0.01]	139.9 (10.1)	3.5 (03.3)	−3.3 (04.2)
FA	[0.43]	[0.39]	[0.02]	218.9 (10.6)	101.8 (05.8)
PH	[0.43]	[0.49]	[−0.02]	[0.38]	323.0 (13.7)
**SEX**					
AC	234.1 (09.0)	87.1 (04.4)	−17.0 (03.5)	95.9 (04.8)	139.8 (06.2)
SO	[0.38]	222.0 (07.8)	−3.8 (03.4)	85.2 (04.5)	141.2 (06.1)
EM	[−0.09]	[−0.02]	151.3 (10.4)	2.0 (03.4)	−18.7 (04.5)
FA	[0.42]	[0.39]	[0.01]	219.5 (10.6)	99.8 (05.8)
PH	[0.50]	[0.52]	[−0.08]	[0.37]	330.8 (13.9)
**AGE**					
AC	232.3(09.0)	86.8(04.4)	−22.7(03.7)	91.1(04.7)	128.4(06.0)
SO	[0.38]	223.3(07.8)	−6.0(03.6)	83.1(04.5)	135.5(06.0)
EM	[−0.12]	[−0.03]	166.4(10.9)	2.7(03.5)	−10.6(04.5)
FA	[0.41]	[0.38]	[0.01]	213.7(10.5)	95.4(05.6)
PH	[0.47]	[0.50]	[-0.05]	[0.36]	324.1(13.8)
	Error [correlations]				
Pairs	E_16−26_	E_2−17_	E_3−13_		E_10−25_ /E_15−30_
**LANGUAGE**					
Spanish	[.25]	[−0.26]	[0.28]		[0.52] /[0.24]
Brazilian					[0.32] /[0.20]
**SEX**					
Men					[0.43] /[0.25]
Women					[0.34] /[0.23]
**AGE**					
11–12 years old					[0.25] /[0.17]
13–14 years old					[0.38] /[0.23]
15–16 years old					[0.49] /[0.26]
17–18 years old					[0.43] /[0.25]

*AC, Academic; SO, Social; EM, Emotional; FA, Family; PH, Physical. All estimated parameters were statistically significant for α = 0.05. Negatively worded items (3, 4, 8, 12, 13, 14, 18, 22, 23, and 28) were inverted*.

### Reliability

Alpha reliability coefficients for the total scale were 0.86 in the Spanish sample, 0.83 in the Brazilian, 0.84 in men, 0.85 in women, 0.86 in the 11–12 year-old age group, 0.84 in the 13–14 year-old age group, 0.84 in the 15–16 year-old age group, and 0.85 in the 17–18 year-old age group (for factor details, see Table 2).

### Relation to parenting dimensions

The acceptance/involvement dimension of the ESPA29 scale related positively to academic, social, family, and physical self-concept, whereas the strictness/imposition dimension was related negatively with academic, social, emotional, and family self-concept (Table 7). The correlations had a similar effect size to those reported in other studies analyzing the relation between parenting and self-esteem (Felson and Zielinsky, 1989; Barber et al., 1992; Musitu and García, 2001, 2004). It was note that family self-concept correlation with acceptance/involvement was.39 (*r*^2^ = 15%) (Musitu and García, 2001, 2004).

**Table 7:** Correlations and *R*^2^ between five self-concept dimensions with two major parental socialization dimensions.

		**Acceptance/involvement**		**Strictness/imposition**	
	***M (SD)***	***r*[95% CI]**	***R*^2^[95% CI]**	***r*[95% CI]**	***R*^2^[95% CI]**
Academic	6.57(1.96)	0.253[0.226,0.280]	0.06[0.05,0.08]	−0.023[−0.052,0.006]	0.00[0.00,0.00]
Social	7.23(1.46)	0.102[0.073,0.131]	0.01[0.01,0.02]	−0.022[−0.051,0.007]	0.00[0.00,0.00]
Emotional	5.13(1.94)	0.053[0.024,0.082]	0.00[0.00,0.01]	−0.112[−0.141,-0.083]	0.01[0.02,0.01]
Family	7.90(1.71)	0.388[0.363,0.413]	0.15[0.13,0.17]	−0.154[−0.182,-0.125]	0.02[0.03,0.02]
Physical	6.13(1.98)	0.176[0.148,0.204]	0.03[0.02,0.04]	−0.045[−0.074,-0.016]	0.00[0.01,0.00]
	*M (SD)*	3.17(0.442)	1.76(0.379)		

## Discussion

Overall, the results of this study validate the Brazilian version of the AF5 Five-Factor Self-Concept Questionnaire. This study provides support for the AF5's multidimensionality across samples of language, sex, and adolescent age. First, the results from separate analyses for samples of Spanish, Brazilian, men, women, and four adolescent age groups from 11 to 18 years old confirm that the proposed five-dimensional correlated model of the AF5 provide a better fit to the data as compared to competitive one-dimensional and five-orthogonal-dimensional models of self-concept. Second, combined multi-sample nested factor analysis showed that the AF5 multidimensional model is largely invariant across related samples of language (Spanish vs. Brazilian [Portuguese]), sex, and adolescent age. The CFA fully corroborates the theoretical structure of the AF5 Five-Factor Self-Concept Questionnaire, supporting the five dimensions of the self-concept construct proposed in the AF5. Concretely, the three multi-sample CFA analyses demonstrated invariance, fixing the same factor pattern of coefficients, factor covariances and variances, and error variances across the groups, satisfying the prerequisite for meaningful multi-sample comparisons when using the AF5 (Cheung and Rensvold, 2002; Spencer et al., 2005). For Brazilian- and Spanish-speakers, men and women, and adolescents across four age groups (11–12, 13–14, 15–16, and 17–18), the analyses showed that: (a) participants conceptualize the pattern of salient and non-salient loadings in a similar way, (b) participants show equivalent strengths of relations between specific scale items and the underlying construct, (c) the correlations among the factors and the range of diversity of responses given to each item are equivalent across groups, and (d), the results meet the strict test of equal error variances (Byrne, 1994), fully with respect to sex, and partially with respect to language and age samples. Additionally, the reliability for all items and dimensions across the related groups was good, with similar results to those obtained in other studies with this instrument (Tomás and Oliver, 2004; García et al., 2006; García and Musitu, 2009). Overall, the findings provide initial evidence for the proposed five-dimensional factor structure measurement of self-concept among adolescents across language (Brazilian [Portuguese] vs. Spanish), sex (men vs. women), and age groups (11–12, 13–14, 15–16, and 17–18 years old), extending results of currently limited research (Tomás and Oliver, 2004; García and Musitu, 2009; Elosua and Muñiz, 2010; García et al., 2011, 2013).

Our results confirm that the correlated five-factor model of the AF5, consisting of academic, social, emotional, family, and physical self-concept, is preferable to the one-dimensional and five-dimensional orthogonal competitive models. The findings of this study concur with previous research that supports the five-factor model of the AF5, using both exploratory (Marchetti, 1997; Martínez et al., 2003; García and Musitu, 2009) and confirmatory (Tomás and Oliver, 2004; García et al., 2006; García and Musitu, 2009; Murgui et al., 2012) factor analyses. The results also support multidimensional theoretical model on which the AF5 is based (Shavelson et al., 1976). Convergent with this model, all items underlie a common construct; the internal consistency of the eight groups analyzed ranged between 0.83 and 0.86. In fact, when we constrained our data to be consistent with a single one-factor model (e.g., Rosenberg, 1965; Baumeister et al., 2003), goodness-of-fit indexes failed to meet conventional standards, indicating a poorest fit. These results reinforce the multidimensional conceptualization of the AF5, emphasizing that a global estimate of self-concept may hide important evaluative distinctions that people make about their adequacy in diverse domains of their lives (see Marsh et al., 2006; Marsh and O'Mara, 2008; Veiga et al., 2015). It is especially notable that our results support the equivalence of factor loadings and variance-covariance matrices among related samples.

Furthermore, in order to have an external validity index, findings indicate that self-concept is associated with the two main parenting dimensions (i.e., acceptance/involvement strictness/imposition) (Felson and Zielinsky, 1989; Barber, 1990; Musitu and García, 2001; López-Jáuregui and Oliden, 2009; Fuentes et al., 2011a,b). The results show that self-concept is positively related with the acceptance/involvement parenting dimension (e.g., practices of reasoning and warmth) and negatively related with the strictness/imposition parenting dimension (e.g., practices of verbal scolding, physical punishment, and revoking privileges). These results offer theoretical and empirical congruent relations with those reported in other studies that analyze the association between self-concept and parenting (Barry et al., 2008), indicating that high self-concept is more likely to be associated with positive parenting, whereas low self-concept tends to be associated with negative parenting (Lamborn et al., 1991; Steinberg et al., 1994; Calafat et al., 2014; García et al., 2015). The present study found a correlation of.39 (*r*^2^ = 15%) between family-AF5 self-concept and the acceptance/involvement parenting dimension, reinforcing the multidimensional perspective of the self-concept (Shavelson et al., 1976; Marsh, 1993; Marsh et al., 2006).

This article is not without limitations. First, the age samples of the present work are limited to the full adolescent age range that we have analyzed. The present results are important given that adolescence is critical in terms of the development of self-esteem, but future research should also consider a wider range of age samples. Second, our results are linked to two particular languages (Brazilian [Portuguese] and Spanish), but possible differences must be taken into account when generalizing to other countries and cultures. Despite these two main limitations, the present work reinforces the multidimensional structure of self-esteem as conceptualized and measured by the AF5. In line with this conceptualization, all items underlie a common construct, present clear relations of item-factor structure on hypothesized domains of self-esteem, and clear invariance of relations between factors. These results satisfy the prerequisite for meaningful multi-sample comparisons when using the AF5 (e.g., Cheung and Rensvold, 2002; Spencer et al., 2005). Our results showed that the instrument is comprehensive, psychometrically sound, brief, easy to complete, and adequate for the multidimensional assessment of self-concept. Therefore, the Five Factor Self-Concept Questionnaire AF5 can be applied in the adolescence population of Brazil with the validity guarantees that establish the results of the applied analyses.

## Author contributions

FG, IM, NB, EC, OG, and ES had participated in the intellectual content, the analysis of data, and the writing of the work. FG, IM, NB, EC, OG, and ES had reviewed the final version of the work and they approve it for publication.

### Conflict of interest statement

The authors declare that the research was conducted in the absence of any commercial or financial relationships that could be construed as a potential conflict of interest.
